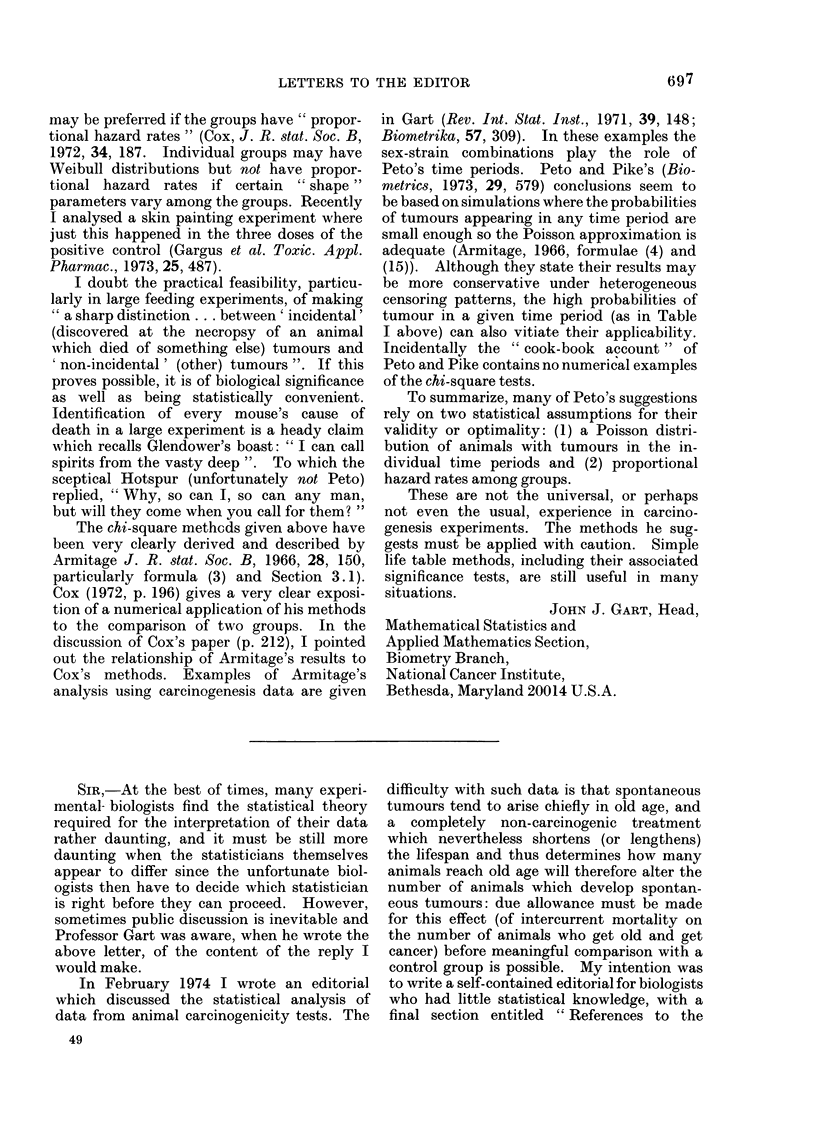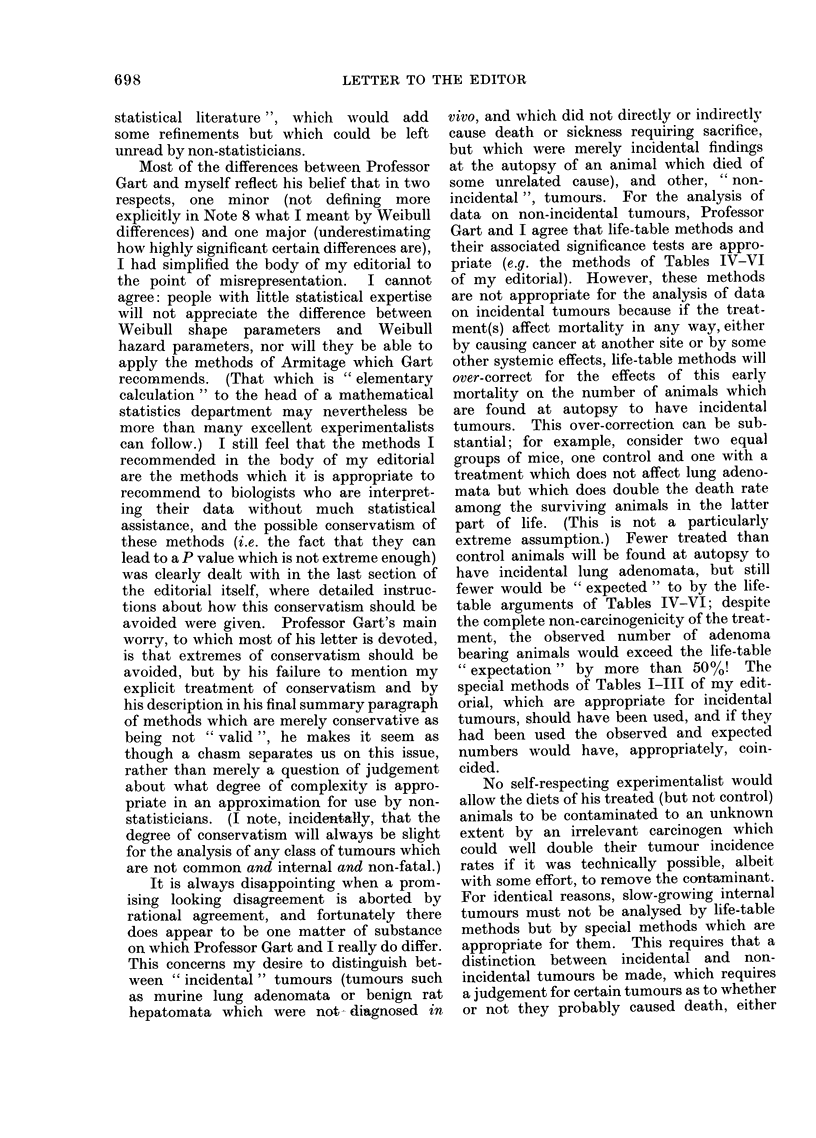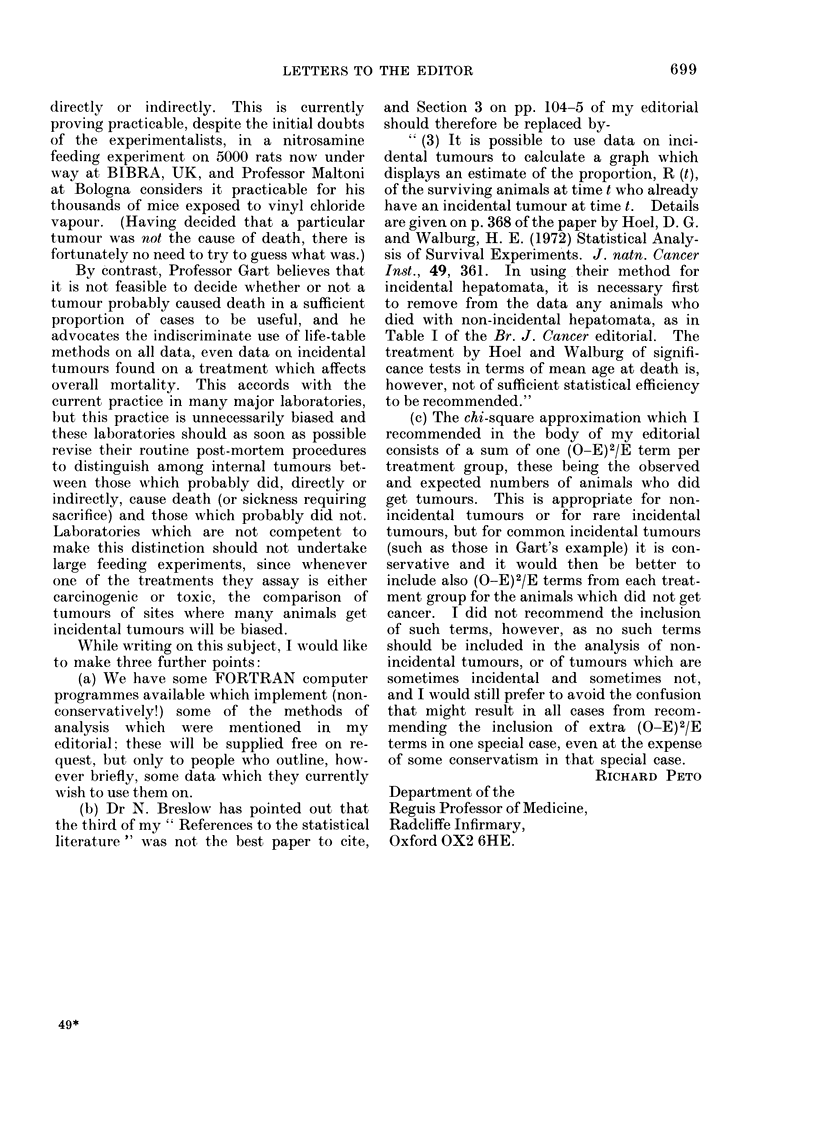# Letters to the Editor

**Published:** 1975-06

**Authors:** Richard Peto


SIR,—At the best of times, many experimental biologists find the statistical theory required for the interpretation of their data rather daunting, and it must be still more daunting when the statisticians themselves appear to differ since the unfortunate biologists then have to decide which statistician is right before they can proceed. However, sometimes public discussion is inevitable and Professor Gart was aware, when he wrote the above letter, of the content of the reply I would make.

In February 1974 I wrote an editorial which discussed the statistical analysis of data from animal carcinogenicity tests. The difficulty with such data is that spontaneous tumours tend to arise chiefly in old age, and a completely non-carcinogenic treatment which nevertheless shortens (or lengthens) the lifespan and thus determines how many animals reach old age will therefore alter the number of animals which develop spontaneous tumours: due allowance must be made for this effect (of intercurrent mortality on the number of animals who get old and get cancer) before meaningful comparison with a control group is possible. My intention was to write a self-contained editorial for biologists who had little statistical knowledge, with a final section entitled " References to the statistical literature ", which would add some refinements but which could be left unread by non-statisticians.

Most of the differences between Professor Gart and myself reflect his belief that in two respects, one minor (not defining more explicitly in Note 8 what I meant by Weibull differences) and one major (underestimating how highly significant certain differences are), I had simplified the body of my editorial to the point of misrepresentation. I cannot agree: people with little statistical expertise will not appreciate the difference between Weibull shape parameters and Weibull hazard parameters, nor will they be able to apply the methods of Armitage which Gart recommends. (That which is " elementary calculation " to the head of a mathematical statistics department may nevertheless be more than many excellent experimentalists can follow.) I still feel that the methods I recommended in the body of my editorial are the methods which it is appropriate to recommend to biologists who are interpreting their data without much statistical assistance, and the possible conservatism of these methods (*i.e.* the fact that they can lead to a $P$ value which is not extreme enough) was clearly dealt with in the last section of the editorial itself, where detailed instructions about how this conservatism should be avoided were given. Professor Gart's main worry, to which most of his letter is devoted, is that extremes of conservatism should be avoided, but by his failure to mention my explicit treatment of conservatism and by his description in his final summary paragraph of methods which are merely conservative as being not " valid ", he makes it seem as though a chasm separates us on this issue, rather than merely a question of judgement about what degree of complexity is appropriate in an approximation for use by non-statisticians. (I note, incidentally, that the degree of conservatism will always be slight for the analysis of any class of tumours which are not common *and* internal *and* non-fatal.)

It is always disappointing when a promising looking disagreement is aborted by rational agreement, and fortunately there does appear to be one matter of substance on which Professor Gart and I really do differ. This concerns my desire to distinguish between " incidental " tumours (tumours such as murine lung adenomata or benign rat hepatomata which were not diagnosed *in vivo*, and which did not directly or indirectly cause death or sickness requiring sacrifice, but which were merely incidental findings at the autopsy of an animal which died of some unrelated cause), and other, " non-incidental ", tumours. For the analysis of data on non-incidental tumours, Professor Gart and I agree that life-table methods and their associated significance tests are appropriate (*e.g.* the methods of Tables IV–VI of my editorial). However, these methods are not appropriate for the analysis of data on incidental tumours because if the treatment(s) affect mortality in any way, either by causing cancer at another site or by some other systemic effects, life-table methods will *over*-correct for the effects of this early mortality on the number of animals which are found at autopsy to have incidental tumours. This over-correction can be substantial; for example, consider two equal groups of mice, one control and one with a treatment which does not affect lung adenomata but which does double the death rate among the surviving animals in the latter part of life. (This is not a particularly extreme assumption.) Fewer treated than control animals will be found at autopsy to have incidental lung adenomata, but still fewer would be " expected " to by the life-table arguments of Tables IV–VI; despite the complete non-carcinogenicity of the treatment, the observed number of adenoma bearing animals would exceed the life-table " expectation " by more than 50%! The special methods of Tables I–III of my editorial, which are appropriate for incidental tumours, should have been used, and if they had been used the observed and expected numbers would have, appropriately, coincided.

No self-respecting experimentalist would allow the diets of his treated (but not control) animals to be contaminated to an unknown extent by an irrelevant carcinogen which could well double their tumour incidence rates if it was technically possible, albeit with some effort, to remove the contaminant. For identical reasons, slow-growing internal tumours must not be analysed by life-table methods but by special methods which are appropriate for them. This requires that a distinction between incidental and non-incidental tumours be made, which requires a judgement for certain tumours as to whether or not they probably caused death, either directly or indirectly. This is currently proving practicable, despite the initial doubts of the experimentalists, in a nitrosamine feeding experiment on 5000 rats now under way at BIBRA, UK, and Professor Maltoni at Bologna considers it practicable for his thousands of mice exposed to vinyl chloride vapour. (Having decided that a particular tumour was *not* the cause of death, there is fortunately no need to try to guess what was.)

By contrast, Professor Gart believes that it is not feasible to decide whether or not a tumour probably caused death in a sufficient proportion of cases to be useful, and he advocates the indiscriminate use of life-table methods on all data, even data on incidental tumours found on a treatment which affects overall mortality. This accords with the current practice in many major laboratories, but this practice is unnecessarily biased and these laboratories should as soon as possible revise their routine post-mortem procedures to distinguish among internal tumours between those which probably did, directly or indirectly, cause death (or sickness requiring sacrifice) and those which probably did not. Laboratories which are not competent to make this distinction should not undertake large feeding experiments, since whenever one of the treatments they assay is either carcinogenic or toxic, the comparison of tumours of sites where many animals get incidental tumours will be biased.

While writing on this subject, I would like to make three further points:

(a) We have some FORTRAN computer programmes available which implement (non-conservatively!) some of the methods of analysis which were mentioned in my editorial; these will be supplied free on request, but only to people who outline, however briefly, some data which they currently wish to use them on.

(b) Dr N. Breslow has pointed out that the third of my " References to the statistical literature " was not the best paper to cite, and Section 3 on pp. 104–5 of my editorial should therefore be replaced by-

" (3) It is possible to use data on incidental tumours to calculate a graph which displays an estimate of the proportion, R ($t$), of the surviving animals at time $t$ who already have an incidental tumour at time $t$. Details are given on p. 368 of the paper by Hoel, D. G. and Walburg, H. E. (1972) Statistical Analysis of Survival Experiments. *J. natn. Cancer Inst.*, **49**, 361. In using their method for incidental hepatomata, it is necessary first to remove from the data any animals who died with non-incidental hepatomata, as in Table I of the *Br. J. Cancer* editorial. The treatment by Hoel and Walburg of significance tests in terms of mean age at death is, however, not of sufficient statistical efficiency to be recommended."

(c) The *chi*-square approximation which I recommended in the body of my editorial consists of a sum of one $(O-E)^2/E$ term per treatment group, these being the observed and expected numbers of animals who did get tumours. This is appropriate for non-incidental tumours or for rare incidental tumours, but for common incidental tumours (such as those in Gart's example) it is conservative and it would then be better to include also $(O-E)^2/E$ terms from each treatment group for the animals which did not get cancer. I did not recommend the inclusion of such terms, however, as no such terms should be included in the analysis of non-incidental tumours, or of tumours which are sometimes incidental and sometimes not, and I would still prefer to avoid the confusion that might result in all cases from recommending the inclusion of extra $(O-E)^2/E$ terms in one special case, even at the expense of some conservatism in that special case.

RICHARD PETO

Department of the
Reguis Professor of Medicine,
Radcliffe Infirmary,
Oxford OX2 6HE.